# Hearing Loss Contributes to Balance Difficulties in both Younger and Older Adults

**DOI:** 10.21767/2572-5483.100033

**Published:** 2018-04-09

**Authors:** Victoria Kowalewski, Rita Patterson, Jessica Hartos, Nicoleta Bugnariu

**Affiliations:** University of North Texas Health Science Center, USA

**Keywords:** Postural control, Hearing loss, Balance, Older adults, Hearing aids, Simulated hearing loss, Falls

## Abstract

**Objective:**

The number of steps required to regain balance is an easily obtainable clinical outcome measure. This study assessed whether number of steps during loss of balance could identify older adults with hearing loss who have balance deficits. We aimed to answer two questions: 1) Does hearing loss negatively affect the ability to regain balance, as reflected by an increased number of steps needed to respond to a perturbation while simultaneously attending to speech-in-noise; and 2) Do hearing aids improve balance control, reflected by a decrease in number of steps needed to regain balance?

**Methods:**

20 young adults and 20 older adults with normal hearing, and 19 older adults with hearing loss performed an auditory-balance dual-task. Participants were asked to listen and repeat back sentences from a standardized audiology test, while simultaneously responding to backward surface translations. Outcome measures were performed on the auditory test and number of steps needed to regain balance. Repeated measures ANCOVA models were run in using group, time, hearing levels, and perturbation levels as predictors.

**Results:**

Auditory scores confirmed difficulty hearing speech-in-noise in older adults with hearing loss and no hearing aids, and in young and older adults with normal hearing and simulated hearing loss. Results showed that group, auditory and balance conditions are significantly related to both outcomes measures and time is not significant for steps. Older adults with hearing loss had a significant increase in number of steps needed to regain balance compared to young adults and older adults with normal hearing.

**Conclusion:**

Number of steps may be an appropriate clinical assessment tool for identifying fall risk in older adults with hearing loss. Further research needs to be performed to identify proper assessments and treatment interventions for older adults with hearing loss who have balance deficits.

## Introduction

Falls are a common problem with adults 65 years and older. One-third of older adults fall annually, costing the United States government approximately 34 billion dollars to cover direct medical expenses for procedures and hospitalization [1,2]. Falls are not only financially costly; falls also burden families taking care of the older adult, stress the constantly shrinking budget for Medicare, decrease the quality of life for the older adult, and may even lead to death of the older adult [3,4].

Another common problem plaguing older adults is hearing loss. Age-related hearing loss affects greater than 60% of people aged 70-79 and 80% of those 80 and older [5]. In the USA, hearing loss has expanded at a rate of 160% of the total population growth and continues to grow due to an aging population [6,7]. Evidence now suggests older adults should address hearing loss because untreated hearing loss may have consequences such as depression, cognitive impairment, and even dementia [8–10].

Moreover, recent evidence has also linked hearing loss to balance deficits in older adults through fall-risk associated assessments, such as slower walking speed and poor Romberg scores [11,12]. These balance deficits increase when noise is present during balance testing [13]. Although the mentioned evidence highlights the need to identify older adults with hearing loss who are at risk for falling, to our knowledge no study has investigated the impact of hearing loss on ability to regain balance following an unexpected loss of balance. Number of steps is an observable clinical outcome measure that can be used when administering reactive balance tests, such as the Nudge Test, to identify an older adult faller [14,15]. An increased number of recovery steps after an unexpected loss of balance are associated with an increased risk for falling [16–18].

We aimed to answer two questions: 1) does hearing loss negatively affect the ability to regain balance as reflected by an increased number of steps needed after a perturbation, and 2) do hearing aids reverse this effect and improve balance control, reflected by a decrease in number of steps needed to regain balance.

We hypothesize older adults with hearing loss will take a greater number of steps during an unexpected loss of balance, compared to young adults with normal hearing and older adults with normal hearing.

## Methods

Twenty-five young adults, 33 healthy older adults with normal hearing, and 22 older adults with hearing loss were verbally informed about the research study and voluntarily agreed to participate through Institutional Review Board (IRB)-approved informed consent. All participants were phone screened prior to enrollment to ensure no visual, vestibular, somatosensory, auditory, health conditions or balance impairments existed that would restrict ability or confound results of the study. Participants were excluded if they had a history of motion sickness/dizziness, or were currently taking medications that affect balance.

Participants underwent cognitive and sensory screening to ensure no undiagnosed cognitive or sensory impairments were present. Five young adults, 13 older adults with normal hearing, and three older adults with hearing loss were excluded due to either undiagnosed cognitive or sensory impairments, or withdrew from the study; resulting in a final count of 20 young adults, 20 older adults with normal hearing, and 19 older adults with hearing loss who participated.

Participants underwent dual-task auditory and balance testing while standing on an instrumented dual-belt treadmill. Participants were required to stand and maintain their balance with unexpected surface translations while simultaneously listening and repeating back sentences. Dual-task auditory- balance sentences were randomized to control for a learning effect [19–21]. Participants were required to listen and repeat sentences from the standardized audiology outcome measure, the Bamford-Kowal-Bench Speech-In-Noise (BKB-SIN) test [22]. These are simple sentences like “the football player lost a shoe.” Each sentence has a specific speech-to-noise ratio, and the scoring on the BKB-SIN outcome measure indicates the required ratio of speech-in-noise for a participant to be able to correctly repeat back 50% of the sentences. The higher the BKB-SIN score, the lower the performance on the auditory test. There were three auditory conditions: 1) no audio sound, no repeat back resulting in the single task of maintaining balance; 2) normal hearing condition in which the BKB-SIN audio files were played, and participants with a diagnosis of hearing loss wore their hearing aids; and 3) hearing loss condition in which the audio files were manipulated to simulate hearing loss for the young and old adults without a hearing loss diagnosis, and participants with a hearing loss diagnosis performed the task without their hearing aids. Participants with hearing loss received the audio input through the speakers, which was delivered directly to the ear via hearing aids. In order to standardize audio input directly to the ear, participants with normal hearing received the audio input to the ear through Bose® QuietComfort 35 wireless headphones.

Backward surface translations were delivered through the treadmill dual-belt system causing the participant to experience a forward loss of balance, while he or she simultaneously listened and repeated the sentence. Three balance conditions were delivered: “0” at 0 m/s^2^ and no backward surface translations, resulting in the single task of listening and repeating back the sentence; “1” backward surface translations at acceleration of 2 m/s^2^; and “2” backward surface translations at acceleration of 5 m/s^2^. The surface translations induced a loss of balance requiring the participants to take 1 or more compensatory steps forward to maintain their balance. An overhead harness system equipped to support up to 181 kg was in place to prevent participants from hitting the ground if a fall would occur.

Combinations of three auditory and three balance conditions were provided randomly and each participant completed 8 trials per combination of auditory-balance conditions.

**Figure 1:**
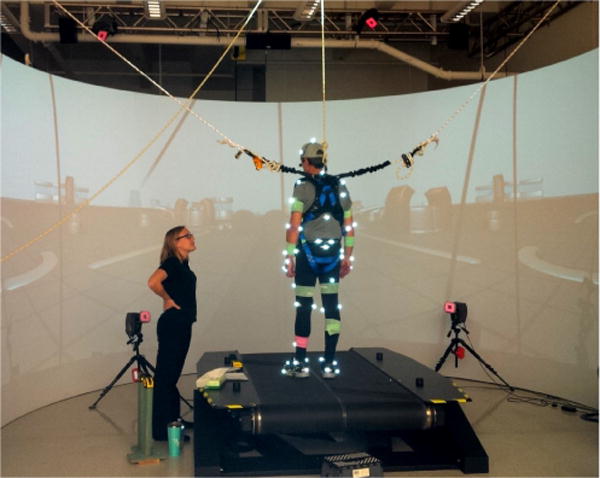
An example image of the research study performed in the laboratory. The participant is standing on a dual-belt treadmill and wearing 54 reflective markers that are being captured by 12 surrounding cameras.

A 12 camera Motion Analysis System collected kinematic data from 54 reflective markers placed on anatomical landmarks of the body. The V-gait treadmill system by Motek Medical containing 2 separate force plates mounted underneath each belt was used to deliver surface translations and record force data (Figure 1).

The primary outcome measures were number of steps and BKB-SIN scores. Number of steps was recorded using visual observation with Cortex Motion Analysis to verify for any uncertainties. Only 1 fall into the harness occurred during data collection; therefore, steps leading to the fall were counted. The BKB-SIN was scored by a single grader, who wore headphones connected via Bluetooth to a microphone worn by the participant.

The number of steps and BKB-SIN scores across the 8 trials per combination of auditory-balance conditions were averaged by person, resulting in an average outcome score per combination. For each outcome, repeated measures ANCOVA models were run in Stata 13.1 using group, time, auditory condition, and balance condition as predictors. All independent variables were coded as categorical with the first group entered as the referent group, and time, auditory, and balance conditions designated as repeated measures variables. The between-participants error terms was designated as “ID|time”, ID was designated the variable representing the lowest unit in the between-participants error term, and time was designated as the group variable for computing the pooled covariance matrix.

## Results

Baseline characteristics for the sample of 59 participants by group are presented in Table 1. Young adults with normal hearing (YANH), Older adults with normal hearing (OANH) and Older adults with hearing loss (OAHL).

**Figure 2:**
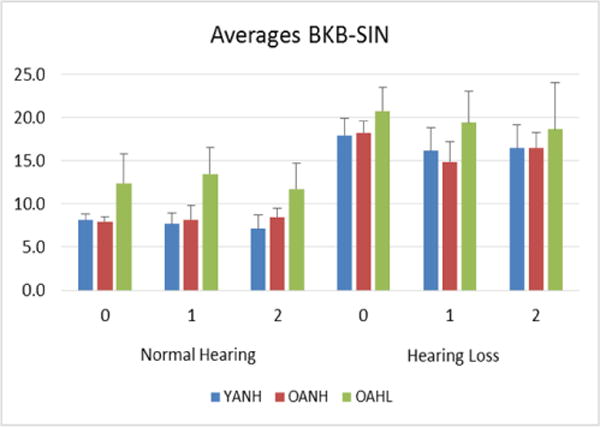
Older adults with hearing loss have significantly higher average BKB-SIN scores, with a higher score indicating worse performance, compared to young adults and older adults with normal hearing during Level 0, 1, and 2 surface translations. All adults perform significantly worse under the hearing loss condition. YANH=Young Adults with Normal Hearing; OANH=Older Adults with Normal Hearing; OAHL=Older Adults with Hearing Loss. Normal Hearing=Normal Hearing/Hearing Aid condition; Hearing Loss=Simulated Hearing Loss/No Hearing Aid condition.

The results suggest that group, auditory condition and balance condition (perturbation level) are significantly related to both outcome measures. There were significant difference in the BKB-SIN score between groups, auditory and balance conditions (all p<0.0001) (Figure 2).

**Figure 3:**
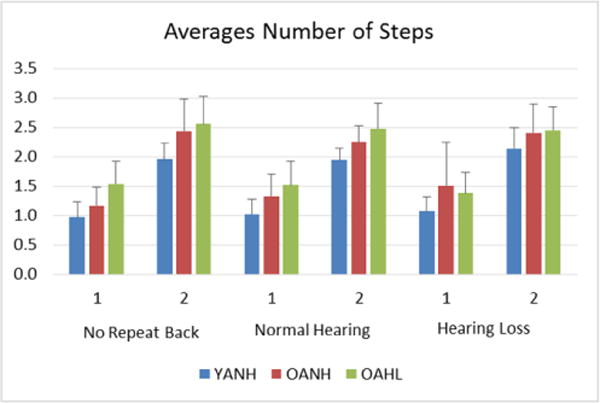
Older adults with hearing loss take a significantly greater number of steps on average compared to young and older adults with normal hearing during no repeat back, normal hearing, and hearing loss conditions, significantly increasing as perturbation level increases from 1 to 2. Number of steps significantly changes across all groups as challenge of task increases from single-task, no repeat back to dual-task condition under normal hearing to dual-task condition under hearing loss. YANH=Young Adults with Normal Hearing; OANH=Older Adults with Normal Hearing; OAHL=Older Adults with Hearing Loss. Normal Hearing=Normal Hearing/Hearing Aid condition; Hearing Loss=Simulated Hearing Loss/No Hearing Aid condition.

There was a significant difference in number of steps between groups, (p=0.0001), auditory conditions (p=0.0301), and balance conditions (p<0.0001) (Figure 3). In addition, the perturbation level has a greater impact on steps, and auditory condition has a greater impact on BKB-SIN. Time was not significant for number of steps (p=0.1828), but was significant for BKB-SIN (p=0.0001), meaning that repeated trials lead to different performances.

## Discussion

The results of this study state there are significant differences in BKB-SIN scores and number of steps between young and older adults with normal hearing, and older adults with hearing loss. These results suggest older adults with hearing loss have poorer reactive balance compared to young and older adults with normal hearing. In older adults with normal hearing, simulated hearing loss negatively affects the ability to regain balance as reflected by an increased number of steps needed after a perturbation. However, the balance performance, as measured by the number of steps required to regain balance while wearing hearings aids, may not have significantly improved enough to prevent a fall. This suggests that while, hearing aids are beneficial for speech recognition, their impact in reversing the negative effect and improve balance control is not as easily measured or understood.

These results coincide with the mixed literature regarding hearing loss and balance difficulty among older adults, as well as whether hearing aids improve balance for older adults with hearing loss [23]. Older adults with hearing loss have been shown to have increased sway compared to older adults with normal hearing, and hearing aids have been shown to improve static balance and balance outcome measures such as the Berg Balance Scale (BBS) [12,24,25]. Older adults with hearing loss have also been shown to have no difference in performance on physical tasks and outcome measures, such as the Timed-Up-And-Go (TUG), and hearing aids did not improve physical function [26,27].

One limitation to the study was the size of the treadmill and the harness system. All individuals were limited in the number and direction of steps able to be taken compared to a setting where participants are able to move more freely [28]. Another limitation is the variably of BKB-SIN scores, particularly among older adults with hearing loss. Some older adults with hearing loss scored close to older adults with normal hearing on the BKB-SIN, while others experienced the floor effect with the BKB-SIN – with higher scores indicating worse performance – and could only attend to a small handful of sentences. The BKB-SIN was designed and is usually administered in a sitting position in a sound-proof booth. The test may have a floor or ceiling effect that has yet to be examined while participants are standing and interacting in a ‘real-world’ setting [29]. The older adults with hearing loss experiencing the floor effect on the BKB-SIN may not have demonstrated a true listening-auditory dual-task based on their hearing ability and these results could actually mask this population at risk for falling, especially in noisy environments [30]. Lastly, many older adults with hearing loss read lips, but the role of vision on speech perception while performing a balance test was not able to be assessed based on the nature of the BKB-SIN [31,32].

It is currently unknown how and why older adults with hearing loss fall more often compared to older adults with normal hearing [33]. More research needs to be performed in order to determine reasons behind why older adults with hearing loss fall more often in order to create proper assessment and treatment strategies for older adults with hearing loss who are at risk for falling [34].

## Conclusion

Older adults with hearing loss appear to require an increased number of steps to regain balance and may be at a greater risk for falling compared to older adults with normal hearing. Number of steps may be an appropriate balance outcome measure to assess fall risk for older adults with hearing.

## Figures and Tables

**Table 1:** Baseline characteristics of young adults with normal hearing, older adults with normal hearing and older adults with hearing loss.

Baseline Characteristics	YANH	OANH	OAHL
Number of Participants (n)	20	20	19
Age (yrs) (mean ± SD)	27.2 ± 3.0	68.7 ± 4.3	73.2 ± 9.1
Height (cm) (mean ± SD)	170.4 ± 8.8	163.6 ± 8.2	169.4 ± 9.0
Weight (kg) (mean ± SD)	74.1 ± 11.5	70.5 ± 19.0	74.0 ± 16.6
Gender (%)			
Male	55%	25%	55%
Female	45%	75%	45%
Race (%)			
White	65%	95%	95%
Asian	25%	5%	0%
Black	10%	0%	5%
Initial HL diagnosis (yrs)			52.3 ± 22.5

YANH=Young Adults with Normal Hearing; OANH=Older Adults with Normal Hearing; OAHL=Older Adults with Hearing Loss
